# Patient safety awareness among 309 surgeons in Enugu, Nigeria: a cross-sectional survey

**DOI:** 10.1186/s13037-019-0216-2

**Published:** 2019-10-25

**Authors:** Arinze Duke George Nwosu, Fidelis Anayo Onyekwulu, Elias Chikee Aniwada

**Affiliations:** 1 Department Of Anaesthesia, National Orthopaedic Hospital, Enugu, Nigeria; 2 Department of Anaesthesia, College Of Medicine, University Of Nigeria, Nsukka, Nigeria; 3 Department of Community Medicine, College Of Medicine, University Of Nigeria, Nsukka, Nigeria

**Keywords:** Adverse healthcare event, Medical error, Patient safety, WHO surgical safety checklist, Wrong-site surgery

## Abstract

**Background:**

Adverse healthcare events are major public health problem with the heaviest burden in the low and middle-income countries. Patient safety awareness among healthcare professionals is known to impact this outcome; thus we set out to appraise the patient safety awareness among surgeons in Enugu, Nigeria.

**Methods:**

A multi-institutional cross-sectional survey was carried out among surgeons in Enugu, Nigeria and data obtained were analyzed using the statistical package for scientific solutions (SPSS) version 20 software.

**Results:**

A total of 309 surgeons were surveyed. Majority of the surgeons (51.9%) had poor perception of patient safety issues. One hundred and twenty respondents (38.8%) have awareness of any institutional protocol for preventing wrong-site surgery while only 35 respondents (11.3%) regularly practiced an institutional protocol for preventing wrong-site surgery. The professional status of the surgeons and years in service showed significant association with perception of patient safety issues.

**Conclusion:**

The patient safety awareness and practice among the surgeons in Enugu, Nigeria is apparently low and this was found to be influenced by the professional status and years in service of the surgeon.

## Background

The term and concept of ‘patient safety’ originated from the pioneering effort of The Anesthesia patient safety foundation (APSF) in 1985. Patient safety may be defined as freedom from untoward events arising from healthcare processes such as may resulting in permanent injury, increased length of stay in hospital or even death, and is thus regarded as a cornerstone of quality care. Harm from adverse healthcare events resulting from medical errors are well documented with recent estimates [1, 2] put at contributing to 200,000 deaths annually in the United States of America alone, beside permanent disability and prolonged hospitalization of the victims and thus represents a major public health problem. A number of studies [3–5] have described an adverse event rate of 4–17% in hospitals in parts of the globe, with two-thirds of the annual global burden of 43 million adverse events occurring in low and middle- income countries (LMICs) [6]. Regrettably, literature regarding patient safety awareness and practice among surgeons in Nigeria, and indeed sub-saharan Africa remains scanty. Beyond the hospitals, adverse healthcare events also occur on outpatient and homecare settings, contributing to harm in health care [7]. Patient safety issues include wrong-site surgery, hospital-acquired infections (HAI), retained surgical items, medication errors, diagnostic errors, workforce safety, abuse/overuse of blood transfusion, mismatched transfusions, pressure ulcers, venous thromboembolism, falls, surgical fires, electrocution and diathermy burns, harm from the use of contaminated drugs and devices, discharge of infants to wrong persons, patient abduction or suicide while under care, among others. Sadly, the population-based study by Leape et al. [8] found that more than two-thirds of these adverse events are preventable.

Whereas much of the attention on patient safety had focused on epidemiology of errors and adverse events, much less effort had been devoted to practices that reduce such events. In view of this concern, resolution WHA55.18, adopted by the Health Assembly in 2002, urged member states to pay the closest possible attention to the problem of patient safety and to establish and strengthen science-based systems necessary for improving patients’ safety and the quality of health care.

A safety culture or climate involving the patient, healthcare professionals and the institutions is advocated to reduce the risk of adverse events relating to exposure to medical care and Zohar et al. [9] have linked the safety culture in different types of healthcare organizations to patient safety outcome.

Medical errors are difficult to measure owing to inadequate reporting and varied definitions, this being further compounded by the fact that most incidents of medical error are not single acts, but rather a chain of events [10]. It is thus instructive that among the greatest advances in patient safety is the growing recognition that medical errors are primarily the result of bad systems, rather than bad individuals. In spite of the traditional autonomy among physicians, there is increasing understanding that engaging other caregivers, patients, and families in patient safety issues helps to raise awareness and prevent medical errors. In tandem with this new paradigm, medical education reform regarding patient safety in medical schools and across the continuum of training and practice has been recommended as one of five fundamental concept areas of healthcare; with the core goal of preparing health professionals’ awareness, skills, commitment and practical training about the scientific pursuit of safer care, through culture change [11].

### Patient safety in surgery

Meara et al. [12] in their recent global surgery survey had found that about 30% of the global burden of disease requires either surgical care or anesthesia management, or both; whereas mortality and morbidity from surgical conditions have increased in LMICs where development in safe surgery and anesthesia have stagnated or regressed. In view of the rapidly expanding volume of surgery [13, 14] with estimate of over 300 million major surgeries performed annually worldwide, the need for safe surgery initiatives becomes very germane. In a systematic review involving 74, 485 patients, de Vries et al. [15] found 9.2% incidence of in-hospital adverse events, with 43.5% being normally preventable, and the majority of events occurring in the operating room. In recognizing the magnitude of this preventable menace to public health, the “WHO surgical safety checklist” was introduced.

Various protocols had earlier been successfully employed by anesthetists and surgeons to improve patient safety through enhanced teamwork and effective communication, resulting in reduced mortality and morbidity in the surgical patient; among them are the Anesthesia patient safety foundation “minimal intra-operative monitoring” in 1986, Canadian orthopedic association “operate through your initials” in 1994 and the American academy of orthopedic surgeons “sign your site” in 1997 and the Joint commission’s “universal protocol” of 2004. By 2008, the “WHO surgical safety checklist” was introduced. Inter-professional checklist briefings generally promoted collaborative team communication and reduced surgical errors [16] and there is abundance evidence that checklist-based patient safety programs significantly reduces complications and death from surgery in diverse clinical settings and institutions around the world [17–19].

Owing to the disproportionate burden of adverse healthcare events in LMICs [6], we sought to evaluate patient safety awareness and practice among physicians who are leaders of the healthcare team as this could be a contributory factor to the dismal statistics of adverse healthcare events in the region. On account of his role, the surgeon’s awareness of patient safety would impact on the patient safety culture of the surgical environment. Consequently this study sought to appraise the surgeons’ consideration of wrong-site surgery, medication errors, hospital-acquired infections, retained surgical items, and over-use/abuse of blood transfusion as patient safety issues.

## Methods

A multi-institutional cross-sectional survey was carried out on all professional cadres of physicians in all the surgical specialties in three tertiary health institutions in Enugu, Nigeria using a self-administered, anonymous, pretested questionnaire. Convenience sampling technique was used to recruit the participants, as effort was made to recruit all the surgeons that were accessible during the period of the study. All 309 respondents were included in the analysis. The questionnaire was divided into sections and comprised a series of items pertaining to socio-demographic and practice characteristics, awareness of patient safety issues, personal encounter with wrong-site surgery, and the knowledge and practice of any institutional protocol for preventing wrong-site surgery. The practice of institutional protocol for preventing wrong-site surgery was evaluated using a 5-point likert scale. Categorical variables are presented as frequency (%).Where relevant results are expressed as mean with standard deviation and median with inter-quartile range.

Statistical analysis was done using SPSS version 20. The chi square test was used to determine association between the respondent’s demographics and appreciation of patient safety issues, knowledge of any institutional protocol for preventing wrong-site surgery and likelihood of encounter with wrong-site surgery. Any association is deemed statistically significant when *p* ≤ 0.05. Multivariate analysis was applied where necessary by determining the adjusted odds ratio (AOR) with 95% confidence interval.

## Results

The respondents are 309 physicians in the various surgical specialties with age ranging between 24 yrs. and 65 yrs. and practice experience ranging between 1 yr and 35 yrs.

Table 1, Shows that majority of the respondents were males; 225(82.5%). Most respondents have practiced for ≤10 yrs.; 245(79.3%). They were mainly resident doctors; 215(69.6%). The median years in practice was 8.70 yrs., with interquartile range; 5–10 yrs. The mean age of the respondents was 35.89 ± 6.88 yrs.

**Table 1:** Socio-demographics of respondents

Socio-demographics	Frequency (n)	Percentage
Age in years		
Mean (SD)	35.89 (6.88)	
Gender		
Male	255	82.5
Female	54	17.5
Professional status		
Consultant/specialist	45	14.6
Resident doctor	215	69.6
Intern/House officer	49	15.9
Years in practice		
≤ 10 years	245	79.3
> 10 years	64	20.7
Years of practice		
Mean (SD)	8.70 (6.28)	
Median (IR^a^)	8.70 (5–10)	

*SD* Standard deviation

IR^a^ - Interquartile range

The data in Table 2, depicts the perception of each patient safety item by the respondents. Hospital-acquired infections were considered by the highest proportion of surgeons 197/309 (64%) as being a significant patient safety issue, while the least proportion 133/309 (43%) considered over-use/abuse of blood transfusion as a significant patient safety issue. Only 150 surgeons (48.5%) have good perception of the patient safety issues considered.

**Table 2:** Significant patient safety concern of respondents

Significant patient safety concern	Yes	No
	*n* (%)	*n* (%)
Hospital -acquired infections	197 (63.8)	112 (36.2)
Wrong- site surgery	170 (55.0)	139 (45.0)
Medication errors	175 (56.6)	134 (43.4)
Abuse/overuse of blood transfusion	133 (43.0)	176 (57.0)
Retained surgical items	167 (54.0)	142 (46.0)
Overall perception of patient safety issues	Frequency(n)	Percent (%)
Poor	159	51.5
Good	150	48.5

Only 26 respondents (8.4%) have ever witnessed wrong-site surgery while 120 respondents (38.8%) were aware of an institutional protocol for preventing wrong-site surgery. The practice of institutional protocol for preventing wrong-site surgery was observed regularly by 35 respondents (11.3%); Table 3.

**Table 3:** Reponses on wrong-site surgery by respondents

Wrong-site surgery	*N* = 309	
	Yes	No
	*n* (%)	*n* (%)
Ever witnessed wrong-site surgery	26 (8.4)	283 (91.6)
Awareness of any institutional protocol for preventing wrong-site surgery	120 (38.8)	189 (61.2)
Practice of any institutional protocol for preventing wrong-site surgery	Frequency(n)	Percentage (%)
Never	151	48.9
Rarely	50	16.2
Sometimes	37	12.0
Often	36	11.6
Regularly	35	11.3
Total	309	100%

There was a strong correlation between the professional status of the surgeons and knowledge of any institutional protocol for preventing wrong-site surgery (*p* < 0.001); but no association was found between gender or years in practice and knowledge of an institutional protocol for preventing wrong-site surgery. All 49 house officers (100%) lack knowledge of any institutional protocol for preventing wrong-site surgery; Table 4.

**Table 4:** Relationship between socio-demographics of respondents and awareness of institutional protocol for preventing wrong-site surgery

Socio-demographics	Yes	No	Test statistics	*p* value
	n (%)	n (%)	Χ^2^	
Gender				
Male	99 (39.0)	155 (61.0)	0.028	0.866
Female	20 (37.7)	33 (62.3)		
Professional status				
Consultant/Specialist	21 (46.7)	24 (53.3)		
Resident doctor	98 (46.0)	115 (54.0)	36.913	< 0.001
Intern/House officer	0 (0.0)	49 (100.0)		
Years in practice				
≤ 10 years	91 (37.4)	152 (62.6)	0.847	0.357
> 10 years	28 (43.8)	36 (56.3)		

Table 5, shows that professional status (*p* = 0.007) and years in practice (*p* = 0.028), but not with gender (*p* = 0.814) were significantly associated with the level of perception of patient safety issues by the surgeons.

**Table 5:** Relationship between socio-demographics of respondents and perception of patient safety issues

Socio-demographics	Good	Poor	Test statistics	*p* value
	*n* (%)	*n* (%)	Χ^2^	
Gender				
Male	123 (48.2)	132 (51.8)	0.056	0.814
Female	27 (50.0)	27 (50.0)		
Professional status				
Consultant/specialist	28 (57.8)	19 (42.2)		
Resident doctor	110 (51.2)	105 (48.8)	9.951	0.007
Intern/House officer	14 (28.6)	35 (71.4)		
Years in practice				
≤ 10 years	111 (45.3)	134 (54.7)	4.984	0.028
> 10 years	39 (60.9)	25 (39.1)		
			95% C.I. for AOR	
	AOR	Sig	Lower	Upper
Professional status				
Consultant/specialist	3.466	0.044	1.916	6.640
Resident doctor	2.459	0.010	1.243	4.865
Intern/House officer	1			
Years in practice				
≤ 10 years	0.629	0.176	0.321	1.232
> 10 years	1			

Furthermore, consultants/specialists were about 4 times (AOR 3.5; 95% CI 1.92–6.64) and resident doctors were about 3 times (AOR 2.5; 95% CI 1.24–4.87) more likely to have good perception of patient safety issues than interns/house officers. Those that had worked for ≤10 years were about 0.6 times less likely (AOR 0.63; 95% CI 0.32–1.23) to have good perception of patient safety issues than those that have worked for > 10 years. Further analysis using multivariate modality supported professional status, but not years of service, as the demographic factor determining perception of patient safety by the surgeons.

## Discussion

The surgical safety checklist is one of the tools designed to enhance patient safety through prevention of healthcare-related adverse events; but in a survey of 502 Brazilian orthopaedic surgeons attending the 44th Brazilian congress of orthopedics and traumatology in 2012 [20], as much as 65.3% were unaware of the surgical safety checklist introduced by the WHO in 2009. Similarly, in a cross-sectional study involving surgeons attending a conference in India in 2014, whereas only 57% (81/142) of the surgeons had heard about the WHO surgical safety checklist only 32% (45/142) of them used it in their surgical practice [21]. This statistics mirrors the trend in other low and middle- income countries (LMICs) as shown by abysmally low implementation of the surgical safety checklist, even in referral hospitals in sub-saharan Africa [22]. The appalling picture contrasts with the developed countries as captured in a global survey involving medical professional from 69 countries [23]; in which fewer respondents used the WHO surgical safety checklist in LMICs compared to high income countries (43.5% vs. 83.5%, *p* < 0.001).

Despite widespread and growing concern about over-use of blood products, only 43% of the respondents in this study considered it a significant issue in patient safety. Evidence of overuse, along with the cost and risks of blood transfusions would suggest that improvement in transfusion practices is vital to improving quality and safety of patient care. A recent review of 450 typical in-patient medical, surgical, or trauma transfusion scenarios by a multidisciplinary panel of experts rated allogeneic red blood cell transfusion as appropriate to improve outcome in only 53 of the scenarios (11.8%), inappropriate in 267 (59.3%), and of uncertain benefit in 130(28.9%) patient scenarios [24].

Hospital-acquired infection (HAI) is the most prevalent consequence of unsafe healthcare, and constitutes a major source of adverse healthcare outcomes [25]. A 5-year retrospective survey involving 22,941 in-patients of a tertiary academic hospital in Nigeria, found the prevalence of HAI to be 2.6% (95% CI: 2.4–2.8), with surgical wards having by far the most infections (48.3%) [26]. A systematic review and meta-analysis of 271 articles however posted a much higher prevalence of HAI (15%) in developing countries of which Nigeria is among, with surgical-site infection being the most common [27]. In tandem with this high prevalence; among the patient safety concerns being rated in this study HAI was considered by majority of the surgeons (64%) as being a significant patient safety issue.

The prevalence of medication errors remains high worldwide, with attendant high mortality in diverse healthcare settings [28, 29]. There is indication that the situation is similar in Nigeria where a national survey [30] involving 2386 professionals (doctors, nurses and pharmacists) posted a prevalence rate of 47%; yet only 57% of the surgeons in this study considered medication errors as an important patient safety issue.

Inadvertent retention of surgical items are relatively common sentinel events [31], with estimates put at 1;5500 surgeries [32] and commonly comprising of sponges, towels, needles, guide-wire, catheter, drains etc. It is unimpressive that only 167 respondents in this study (54%) considered this a significant healthcare safety consideration despite the chilling statistics.

Wrong-site surgery is not rare in Nigeria, albeit being under-reported [33, 34] ^.^Whereas 26 respondents in this study (8.4%) have witnessed wrong-site surgery, 189 respondents (61%) affirmed not being aware of any institutional protocol for preventing it, while only 170 respondents (55%) adjudged the “never- event” as a significant patient safety issue. Only 35 out of 309 respondents (11%) regularly implemented any institutional protocol for preventing wrong-site surgery. The WHO surgical safety checklist is among the tools designed to prevent wrong-site surgery and some studies in Nigeria [35, 36] indicate a high level of awareness regarding the WHO surgical safety checklist, among anesthetists, surgeons and peri-operative nurses in the tertiary health institutions. Quite unfortunately compliance rate and knowledge of purpose of the checklist were disproportionately low in the cited studies, with only about 20% of the respondents in one study [35] correctly stating that the purpose is for patient safety.

Surgical safety checklist education and training have been found to remarkably enhance awareness and compliance [37, 38]. In much the same way mandatory institutional policy was found to profoundly enhance awareness about the WHO surgical safety checklist [39, 40]. Neither of these was in operation in the health facilities surveyed. It is however pertinent to reiterate that as it is with checklist compliance, mandatory implementation regarding patient safety issues as mere statutory requirement would not necessarily guarantee benefits without proper education, training and buy-in by healthcare personnel to engender a true culture of safety. In a comparative study of retrospective recorded surgical safety checklist compliance with concurrent direct –observed compliance in a group of regional hospitals, Saturno et al. [41] found that the compliance in the record books was markedly higher than actual compliance (*p* < 0.001) and this phenomenon was widespread in the hospitals and thus unreliable index of surgical safety checklist implementation. Furthermore, the poor appreciation of the array of patient safety issues considered in this study by the surgeons would suggest that knowledge and implementation of patient safety initiatives must go beyond the WHO surgical safety checklist alone; as the latter even when fully implemented would not satisfactorily address the knowledge gap.

Kerfoot et al. [42] had earlier reported limited patient safety knowledge among medical trainees across a broad range of training levels and specialties in the United States of America, with worse scores recorded by residents in the surgical specialties and medical students. In their study, residents from United States medical schools out-performed those from foreign medical schools (*P* = 0.006) and this is thought to have derived from the incorporation of patient safety into the competency training of many United States medical schools, while similar competency is now mandated for all United States residency programs by the Accreditation council for graduate medical education [43]. Whereas univariate analysis of their data showed that patient safety knowledge levels varied significantly by year of training, age and gender; multivariate analysis affirmed year of training, but not age and gender as determinants of patient safety knowledge. In this study bivariate analysis showed that age and gender of the surgeon had no significant influence; instead only the professional status of the surgeons and the years in service of the surgeon had significant association with the level of appreciation of patient safety issues, with the professional status of the surgeons proving to be the only valid determinant of level of patient safety appreciation on multivariate analysis (AOR; 2.459, 95% C.I.; 1.243–4.865). Patient safety awareness and practice is poor among the surgeons surveyed in Enugu, Nigeria. In view of the current trends in the global burden of disease and the preponderance of surgery- related adverse healthcare events [12, 15], it would appear that improved patient safety awareness among the surgeons will enable a knowledge-based integrated approach that can drive the vision and goals of safer care.

Study limitation**:** The convenient sampling technique deployed for recruitment of the respondents would have excluded surgeons in the private and non-academic institutions, and over-represented the characteristics of those in structured academic programs. Similarly those who were too busy with other activities at the time of the survey or declined to participate in the study were not considered.

## Conclusion

The patient safety awareness and practice among the surgeons in Enugu, Nigeria is apparently low and this was found to be influenced by the professional status and years in service of the surgeon. As this deficit would arguably contribute to unsafe healthcare and adverse outcomes educational and institutional measures to improve on this should be considered.

## Data Availability

All data generated or analyzed during this study are included in this published article. The datasets are available from the corresponding author by reasonable request.

## Abbreviations

AOR: Adjusted odds ratio
APSF: Anesthesia patient safety foundation
CI: Confidence interval
HAI: Hospital- acquired infection
IR: Interquartile range
LMICs: Low and middle-income countries
SD: Standard deviation
SPSS: Statistical package for scientific solutions
WHO: World health organization
